# Medical Items

**Published:** 1892-12

**Authors:** 


### *MEDICAL ITEMS.*

The new management of the Chicago *Corpuscle* confidently assumes its task *sans puer*, and hopes at the close to be *sans reprochi.* We pray that it may be better than its French.

The corner-stone of the new Pasteur Institute, New York, has been laid, and the building is expected to be completed by the new year. It will also contain a department for bacteriological study.

We have received from Messrs. William Wood & Co. one of their handsome pictures for doctors' offices. It is a large photograph of Prof. Billroth's clinic in the Vienna Hospital. This and others of the series will appropriately ornament the office of any physician.

MESSRS. LEA & SHEPARD, of Boston, will please accept our thanks for their unique and pretty little calendar, "All Around the Year, 1893." It is printed in colors from new designs by J. Pauline Sunter, with gilt edges, tassels, rings and chain. The designs are quaint and original, and altogether make a very ornamental calendar for all around the year. Price fifty cents.

---

Dr. A. Reeves Jackson, of Chicago, died on the 12th ult. He was President of the College of Physicians and Surgeons of Chicago, and one of the prominent gynecologists of the city. Dr. Jackson was sixty-five years of age, and his death was due to apoplexy. It is said that Dr. Jackson was the original of Mark Twain's character, "the doctor," in "Innocents Abroad."

---

The newest suggestion in the way of congresses is that which emanates from Paris. It is proposed to hold in the French metropolis, in 1893, an international congress composed of medical men, jurists, hygienists, economists and sociologists, for the study of questions relating to prostitution and the propagation of syphilis. With such a medley of contributors the proceedings ought to prove interesting.—*Med. Press.*

---

Of course everybody will attend the World's Fair in Chicago. For the convenience of the doctors (and their families) who will go, Messrs. Charles Truax, Green & Co. will establish at 75 and 77 Wabash avenue, a bureau of information, where visiting members of the profession will find reading rooms, a postoffice department where mail can reach them, a banking department where checks can be cashed, etc. Any assistance that may be necessary will be cheerfully given. Interpreters will also be provided. All these conveniences will be furnished without charge. Cut this out and save it for your trip.

A SECTION of railway surgery of the Pan-American Medical Congress has been organized with Dr. C. W. P. Brock, of Richmond, Virginia, as executive president. A full list of officers has been provided for each of the constituent countries. At the eleventh annual meeting of the Wabash Railway Surgical Association—the first organization of the kind—Dr. C. B. Stevens, of Fort Wayne, was by unanimous resolution requested to prepare a paper on "Organized Railway Surgery," and read the same to the section on railway surgery of the Pan-American Medical Congress. At the same meeting Dr. Hal C. Wyman, of Detroit, offered the following which was unanimously adopted :

*Resolved*, That each member of this Association solicit his congressman to interest himself in legislation in favor of the Pan-American Medical Congress.

---

The College of Physicians of Philadelphia announces that the next award of the Alvarenga Prize being the income for one year of the bequest of the late Senor Alvarenga, and amounting to about one hundred and eighty dollars, will be made on July 14, 1893, provided that an essay, deemed by the Committee of Award to be worthy of the prize, shall have been offered.

Essays intended for competition may be upon any subject in medicine, but cannot have been published, and must be received by the secretary of the college on or before May 1, 1893.

Each essay must be sent without signature, but must be plainly marked with a motto and be accompanied by a sealed envelope having on its outside the motto of the paper and within it the name and address of the author.

It is a condition of competition that the successful essay or a copy of it shall remain in possession of the college ; other essays will be returned upon application within three months after the award.

The Alvarenga prize for 1892 has been awarded to Dr. R. H. L. Bibb, of Saltillo, Mexico, for his essay entitled, "Observations on the Nature of Leprosy."

CHARLES W. DULLES,
Secretary.

